# Sound conditioning strategy promoting paracellular permeability of the blood‐labyrinth‐barrier benefits inner ear drug delivery

**DOI:** 10.1002/btm2.10596

**Published:** 2023-09-10

**Authors:** Xueling Wang, Jiayi Gu, Ke Xu, Baoying Xu, Dehong Yu, Hao Wu

**Affiliations:** ^1^ Department of Otolaryngology‐Head and Neck Surgery, Shanghai Ninth People's Hospital Shanghai Jiao Tong University School of Medicine Shanghai China; ^2^ Ear Institute Shanghai Jiao Tong University School of Medicine Shanghai China; ^3^ Shanghai Key Laboratory of Translational Medicine on Ear and Nose Diseases (14DZ2260300) Shanghai China; ^4^ Materdicine Lab, School of Life Sciences Shanghai University Shanghai China

**Keywords:** blood–labyrinth–barrier, hearing loss, permeability, sound conditioning, tight junctions

## Abstract

The therapeutic effects of pharmaceuticals depend on their drug concentrations in the cochlea. Efficient drug delivery from the systemic circulation into the inner ear is limited by the blood‐labyrinth‐barrier (BLB). This study investigated a novel noninvasive sound conditioning (SC) strategy (90 dB SPL, 8–16 kHz, 2 h sound exposure) to temporally enhance BLB permeability in a controllable way, contributing to maximizing the penetration of pharmaceuticals from blood circulation into the cochlea. Trafficking of Fluorescein Isothiocyanate conjugated dextran and bovine serum albumin (FITC‐dextran and FITC‐BSA) demonstrated that paracellular leakage of BLB sustained for 6 h after SC, providing a controllable time window for systemic administration. Cochlear concentrations of dexamethasone (DEX) and dexamethasone phosphate (DEX‐P), respectively transported by transcellular and paracellular pathways, showed a higher content of the latter one after SC, further confirming the key role of paracellular pathway in the SC‐induced hyperpermeability. Results of high‐throughput RNA‐sequencing identified a series of tight junction (TJ)‐associated genes after SC. The expressions of TJ (ZO‐1) were reduced and irregular rearrangements of the junction were observed by transmission electron microscopy after SC. We further determined the inhibiting role of Rab13 in the recruitment of ZO‐1 and later in the regulation of cellular permeability. Meanwhile, no significant change in the quantifications of endothelial caveolae vesicles after SC indicated that cellular transcytosis accounted little for the temporary hyperpermeability after SC. Based on these results, SC enhances the BLB permeability within 6 h and allows systemically applied drugs which tend to be transported by paracellular pathway to readily enter the inner ear, contributing to guiding the clinical medications on hearing loss.

**Translational Impact Statement**

Blood‐labyrinth barrier (BLB) strongly restricts the efficient drug delivery from the systemic circulation into the inner ear. Sound conditioning (90 dB SPL, 8‐16 kHz, 2 h) temporally enhanced the permeability of the BLB and provided a time window (0‐6 h) for systemically‐applied substances into the cochlea. SC, as a safe and minimally invasive method, has translational and clinical potential for systemic pharmaceutical delivery to treat inner ear disorders.

## 1 INTRODUCTION

A global report on hearing revealed that over 1.5 billion people currently experience various degrees of hearing loss, which could grow to 2.5 billion by 2050 according to the World Health Organization.^1^ Many technological innovations, including optogenetics, hair cell regeneration or gene editing, provide a much broader range of treatment possibilities for hearing loss.^2^ The key challenge facing therapeutic pharmaceuticals, such as growth factors, viral vectors for genetic manipulation, or even stem cells, is the blood‐labyrinth‐barrier (BLB) that isolates the inner ear from the blood. The BLB locates between the vasculature of stria vascularis (SV) and endolymph of the inner ear, restricting entry of most systemically administrated therapeutics into the inner ear. Endothelial cells exhibit two features that underlie the restrictive properties of the physiological barrier: intercellular junctions that prevent paracellular passage between the blood and the inner ear, and low rates of vesicle trafficking that limit endothelial transcellular transport or transcytosis.^3^ In addition to vascular endothelial cells and junctions in between, the BLB comprises pericytes, basement membrane, and perivascular resident macrophage‐like melanocytes (PVM/Ms). Structural associations and interactions between these components control vascular permeability and maintain the integrity of BLB.^4^, ^5^, ^6^

Several external factors can modulate the permeability of the BLB including diuretics,^7^, ^8^ inflammation,^9^, ^10^, ^11^ and acoustic trauma.^9^, ^10^, ^11^ For instance, furosemide can promote the cochlear concentrations of cisplatin and aminoglycosides by elevating BLB permeability, and further increases ototoxic effects when administered concomitantly.^7^, ^12^ Acoustic trauma could increase the permeability of BLB by either damaging the tight junctions (TJs) between adjacent endothelial cells, claudin‐5 and occludin,^13^ or upregulating matrix metalloproteinases (MMP‐2 and ‐9) which are known to be capable of degrading TJ proteins.^14^ Moreover, noise exposure causes activation of PVM/Ms and physical detachment from capillary walls, as well as decreasing production of tight‐ and adherens‐junction proteins.^15^ Acoustic trauma may regulate BLB permeability and the subsequent uptake of drugs indirectly by inducing inflammation.

A variety of interventions has been implemented to reduce the susceptibility to cochlear injury from noise or ototoxicity by exposing the ear to a stressful stimulus.^16^ One of the most common strategies is to expose the cochlea to a nondamaging sound that stimulates the ear's defenses, making the cochlea more resistant to future permanent injuries, which is called “sound conditioning (SC)” or “toughening.” This phenomenon has been confirmed in mice,^17^ guinea pigs,^18^ gerbils,^19^ rats,^20^ and so forth. Notably, young human exposed to low‐intensity music showed a significant reduction of temporary threshold shift.^21^

Based on these, we proposed that SC with a moderate intensity could increase the permeability of BLB and further facilitate cochlear drug delivery. We screened a proper condition of sound stimulus without causing permanent hearing loss. Trafficking of the fluorescent tracers and quantifications of systemically administrated drugs were used to identify the transport pathway of drug into the cochlea. By combining high‐throughput RNA‐sequencing and molecular biology methods, we explored the underlying mechanisms of SC‐induced hyperpermeability of BLB. This study suggests a great potential of SC as a noninvasive therapeutic opportunity for enhancing inner ear drug delivery and provides a novel strategy to treat inner ear diseases.

## 2 MATERIALS AND METHODS

### 2.1 Animals

Male C57BL/6J mice (28 days old) were purchased from the Shanghai Laboratory Center (Shanghai, China). The mice were housed in a temperature‐controlled (20–24°C) room and a 12/12‐h light/dark cycle and had free access to food and drinking water. All animal studies (including the mice euthanasia procedure) were carried out according to the protocols approved by the Shanghai Jiaotong University, School of Medicine.

### 2.2 Cell culture

HEI‐OC1 cell line was generated from the auditory organ of transgenic mouse Immortomouse™ at House Ear Institute (Los Angeles, CA). HEI‐OC1 was maintained in high‐glucose Dulbecco's Modified Eagle's Medium (Gibco) containing 10% fetal bovine serum (Gibco) without antibiotics at 33°C in a humidified atmosphere of 10% CO_2_.

### 2.3 Optimizing the SC

Mice were placed in separate compartments of a cage and were subjected to a single SC of 2 h using an 8–16 kHz octave band noise at an intensity of 80, 90, and 106 decibel sound pressure level (dB SPL) in a calibrated reverberating chamber. The SC stimulus was generated by an MATLAB software. Calibration of the sound conditioner in open field was performed with an acoustimeter.

Permeability experiments were performed using sodium fluorescein (NaFluo, a hydrophilic and low‐molecular weight (MW: 376 Da) dye. Briefly, mice in control and sound‐conditioned groups intravenously received NaFluo (4%, 5 mg/kg) for 10 min. The SV homogenate was obtained by tissue homogenizer and centrifuged at 15,000 rpm for 5 min to keep the supernatant, and then the fluorescence intensity was examined by a microplate reader at excitation/emission wavelengths of 485/535 nm. NaFluo content was calculated from the calibration curve.

### 2.4 Auditory brainstem response tests

Auditory brainstem response (ABR) analyses were performed to evaluate auditory function before and 1, 14 days after SC. Mice were anesthetized with 1% pentobarbital sodium (35 mg/kg). ABR potentials were recorded with a Tucker‐Davis Technologies (TDT) system (System 3 RZ6, Tucker‐Davis Technologies) in a sound‐proof booth. Pure tone stimuli of 4, 5.6, 8, 11.2, 16, 22, and 32 kHz between 0 and 90 dB SPL were delivered through an open‐field microphone. Subcutaneous electrodes collecting signals were inserted at the pinna (recording electrode), vertex (reference electrode), and rump (ground electrode). The lowest intensity level at which no responses were observed in waveforms was determined to be the threshold. Wave‐I amplitude was determined by measuring the voltage difference between the highest value (peak) and the lowest value (trough) for the first ABR wave.

### 2.5 Immunofluorescence

Cochleae or HEI‐OC1 cells were immediately harvested and fixed by 4% paraformaldehyde (PFA) for 2 h or 15 min at room temperature. SV or the basilar membrane was dissected, blocked with donkey serum (8%), and treated with primary antibodies, anti‐desmin (1:200, Abcam) and anti‐F4/80 (1:200, eBioscience), anti‐CtBP2 (1:200, BD‐Biosciences), anti‐Myosin VIIa (1:200, Proteus Biosciences), anti‐Rab13 (1:200, Affinity) and secondary antibodies against rabbit or rat labeled with Alexa 488 or 594 (1:500, Thermo Fisher Scientific). Isolectin GS‐IB4 conjugated to Alexa‐Fluor 647 (1:100, Thermo Fisher Scientific) was applied for 1 h at room temperature. After mounting and capturing pictures by a laser scanning confocal microscope (Leica, SP8), the coverage of pericytes or PVM/Ms, which was displayed as desmin or F4/80 area/Isolectin area, was quantified by ImageJ.^5^

### 2.6 Quantification of synaptic elements

Confocal z‐stacks along discrete regions of the basilar membrane were made with a 63 × oil immersion objective on a confocal microscope (Leica, SP8). A z‐step was used to capture all synaptic structures of two to three hair cells. The number of functional synapses, identified by juxtaposed CtBP2 and GluA2, were manually counted by visualizing the presence of GluA2 colocalization with CtBP2.

### 2.7 Hematoxylin and eosin staining

Midmodiolar sections were stained with hematoxylin and eosin (H&E) and observed with an optical microscope (Leica, DM6) in basal regions. SV area and thickness were measured and calculated by ImageJ software. SV thickness was measured in the midpoint between the attachment of Reissner's membrane and the spiral prominence.

### 2.8 Leakage analysis

Fluorescent tracers, including FITC‐dextran and FITC‐BSA, were respectively used for the evaluation of paracellular and transcellular BLB permeability in control and sound‐conditioned mice (0, 6, and 24 h after SC). FITC‐dextran (MW: 70 kDa, 1 mg/mL, 10 mg/kg) and FITC‐BSA (MW: 68 kDa, 0.2 mg/mL, 5 mg/kg) were injected via tail vein and allowed to circulate for 10 min. After sacrifice and fixation, SV was carefully dissected into apical, middle, and basal turns, and then mounted, and visualized under a laser scanning confocal microscope (Leica, SP8). Microvessels were labeled by GS‐IB4. The mean fluorescence intensity of regions of interest and percentage endothelial‐filled area with FITC‐BSA is calculated via ImageJ.

Moreover, Evans blue (EB) dye was used to monitor intravasation through the blood–brain barrier (BBB). EB (sigma, 2%, m/v) was intravenously injected through tail vain and circulated for 3 min. After cardiac perfusion, images of brain were captured by optical microscope (Leica, DM6).

### 2.9 DEX or DEX‐P concentrations in the inner ear after intraperitoneally injection

The cochlear tissue samples were analyzed for DEX concentrations using an LC–MS/MS system (liquid chromatography/tandem mass spectrometry, Shimadzu, AB SCIEX API400) equipped with an Acquity UPLC BEH C18 (2.1 mm × 50 mm, 1.7 μm) for chromatographic separation. DEX solution (10 mg/kg) was intraperitoneally injected following SC. 0, 6, 12, and 24 h after DEX administration, the mice were sacrificed by cervical dislocation and the inner ear was quickly removed from the skull. Then the samples were rinsed and homogenized in 0.2 mL cold PBS. The homogenate was centrifuged at 12,000 rpm for 20 min, and the supernatant was subsequently used for LC–MS/MS analysis as previously described.^22^

### 2.10 RNA‐sequencing

RNA‐sequencing was performed by Novogene Co., Ltd (China). SV were harvested and total RNAs from control and SC groups were extracted with Trizol reagent. The mass of total RNA ≥0.2 μg and RNA integrity number ≥ 7.0 was subjected to RNA‐Seq. Samples were sequenced by an Illumina NovaSeq 6000. Differential expression analysis of control and SC groups was performed using the DESeq2 R package (1.20.0). Differentially expressed genes (DEGs) with FC value >0.5 or <− 0.5, and a *p*‐value <0.05 were considered significant. Gene ontology (GO) classification and Kyoto Encyclopedia of Genes and Genomes (KEGG) enrichment analysis of DEGs were implemented by the clusterProfiler R package (3.8.1). Each group of tissues was sequenced with three biological replicates.

### 2.11 Quantitative real‐time PCR

Total RNAs of SV from control and SC groups were extracted and the concentrations were evaluated with a NanoDrop. RNAs were reverse transcribed with a Takara PrimeScript RT reagent kit (Takara Biotechnology) according to the manufacturer's instructions. Quantitative real‐time PCR (qRT‐PCR) was performed using a TB Green Premix Ex Taq kit (Takara Biotechnology). Primer sequences were provided in Table 1.

**TABLE 1:** Quantitative real‐time PCR primer sequences.

Gene	Forward primer	Reverse primer
*Tjp1*	CTCCAGTCAGCCCGCAAAG	CAAGACAACATCCCCTTCTTGA
*Ocel1*	GTCCGTGAGGCCTTTTGA	GGTGCATAATGATTGGGTTTG
*Cldn5*	TTAAGGCACGGGTAGCACTC	ATGTTGGCGAACCAGCAG
*Bves*	CCTCTGCACACAGATCTCCA	CAAGGCAGCTGATGGACTTT
*Jam2*	CCAGAACCTGCAGGAAGATAAA	CCACCACACTTCCAGTCATAA
*Rab13*	TGGATGAGGCTTTCAGTTCC	AGTCCGACTCCCTCAGGTCT
*Rap2c*	GGCCATACCGAGCAGATAAAAAC	TGGATCTGGAGGGCCAAAGA
*Mmp2*	CAACGGTCGGGAATACAGCAG	CCAGGAAAGTGAAGGGGAAGA
*Mmp9*	GCTGACTACGATAAGGACGGC	AGGAAGACGAAGGGGAAGACG
*Pdgfrb*	AGGACAACCGTACCTTGGGTGACT	CAGTTCTGACACGTACCGGGTCTC
*Mfsd2a*	AGAAGCAGCAACTGTCCATTT	CTCGGCCCACAAAAAGGATAAT

The expression levels of mRNAs were normalized to that encoding *Gapdh*.

### 2.12 Transfection of siRNA in cultured HEI‐OC1 cells

HEI‐OC1 cells were transfected with small interfering RNAs (siRNAs) duplexes targeting *Rab13* or with scrambled siRNA control using Lipofectamine 2000 reagent (Invitrogen). The siRNAs were commercially provided from the Genepharma company. The sequences were as follows (sense and antisense, respectively):

*Rab13*‐mus‐198, 5′‐GCAGAGGACAACUUCAACATT‐3′ and 5′‐UGUUGAAGUUGUCCUCUGCTT‐3′;

*Rab13*‐mus‐307, 5′‐CUGGCCAAGAACGAUUCAATT‐3′ and 5′‐UUGAAUCGUUCUUGGCCAGTT‐3′;

*Rab13*‐mus‐688, 5′‐CUGACAAGAAGAAGAACAATT‐3′ and 5′‐UUGUUCUUCUUCUUGUCAGTT‐3′;

Negative control, 5′‐UUCUCCGAACGUGUCACGUTT‐3′ and 5′‐ACGUGACACGUUCGGAGAATT‐3′.

After transfection for 48 or 72 h, cells were collected for qRT‐PCR and western blot assays.

### 2.13 In vitro transwell assay

After transfection by negative control or *Rab13*‐mus‐198 siRNA for 48 h, HEI‐OC1 cells were seeded on the inner surface of transwell inserts (12 mm diameter, 0.4 μm pore size, Corning) and form confluent cell monolayers after 12 h. Afterward, FITC‐dextran (MW: 70 kDa, 1 μg/mL, sigma) was added to the upper chambers and incubated at 33°C for 6 h. Then the medium from the lower chambers was collected and MFI at excitation/emission wavelengths of 485/535 nm was detected using a luminescence plate reader.

### 2.14 Western blot analysis

The cochlear lateral wall was lysed with cold RIPA lysis buffer and homogenized for further heating for 10 min at 95°C. Samples were loaded onto 8% sodium dodecyl sulfate‐polyacrylamide gels and electro‐transferred to polyvinylidene fluoride membranes (Bio‐rad laboratories). Membranes were blocked in quick blocker (beyotime) for 1 h at room temperature, and incubated with anti‐ZO‐1 (1:1000, Invitrogen), anti‐occludin (1:1000, Invitrogen), anti‐MFSD2 (1:1000, proteintech), anti‐Caveolin‐1 (CAV‐1; 1:1000, CST), and anti‐Rab13 (1:1000, Affinity) overnight at 4°C. The blots were then exposed to the horseradish peroxidase‐labeled secondary anti‐rabbit antibody (1:1000) for 1.5 h at room temperature. The proteins were visualized using an enhanced chemiluminescence kit (Thermo Fisher Scientific). The band signals were detected using a gel imaging system (Amersham Biosciences). The intensities were quantified using ImageJ software. Glyceraldehyde‐3‐phosphate dehydrogenase (GAPDH) was used as control of protein expression.

### 2.15 Transmission electron microscopy

Transmission electron microscopy (TEM) was used to observe the ultrastructural changes between the strial endothelial cells of control and 0 or 24 h post‐SC mice. Cochleae were collected immediately after cardiac perfusion and fixed in 2.5% glutaraldehyde/0.1 M PB (pH 7.4) at 4°C overnight, followed by another fixation by osmic acid for 2 h at room temperature. Then the samples were dehydrated by graded ethanol and eluted with acetone. Epon 812 resin was used to embed the samples. Sections (60 nm) were cut with a Leica ultracut microtome, stained with 2% Uranyl acetate and lead citrate for 15 min, respectively, and viewed and imaged under the Electron Microscope (Philips CM‐120). The fractions of the angle of TJs and the caveolae vesicle numbers per unit area were calculated.

### 2.16 Statistics

GraphPad Prism (GraphPad Software) was used for statistical analysis. Unpaired student's test and one‐way analysis of variance with Bonferroni's multiple comparisons posttest were used where appropriate. All quantitative data were presented as mean ± standard deviation (SD). The *p*‐values < 0.05 were considered significant.

## 3 RESULTS

### 3.1 Screening for the optimal condition of sound

**FIGURE 1:**
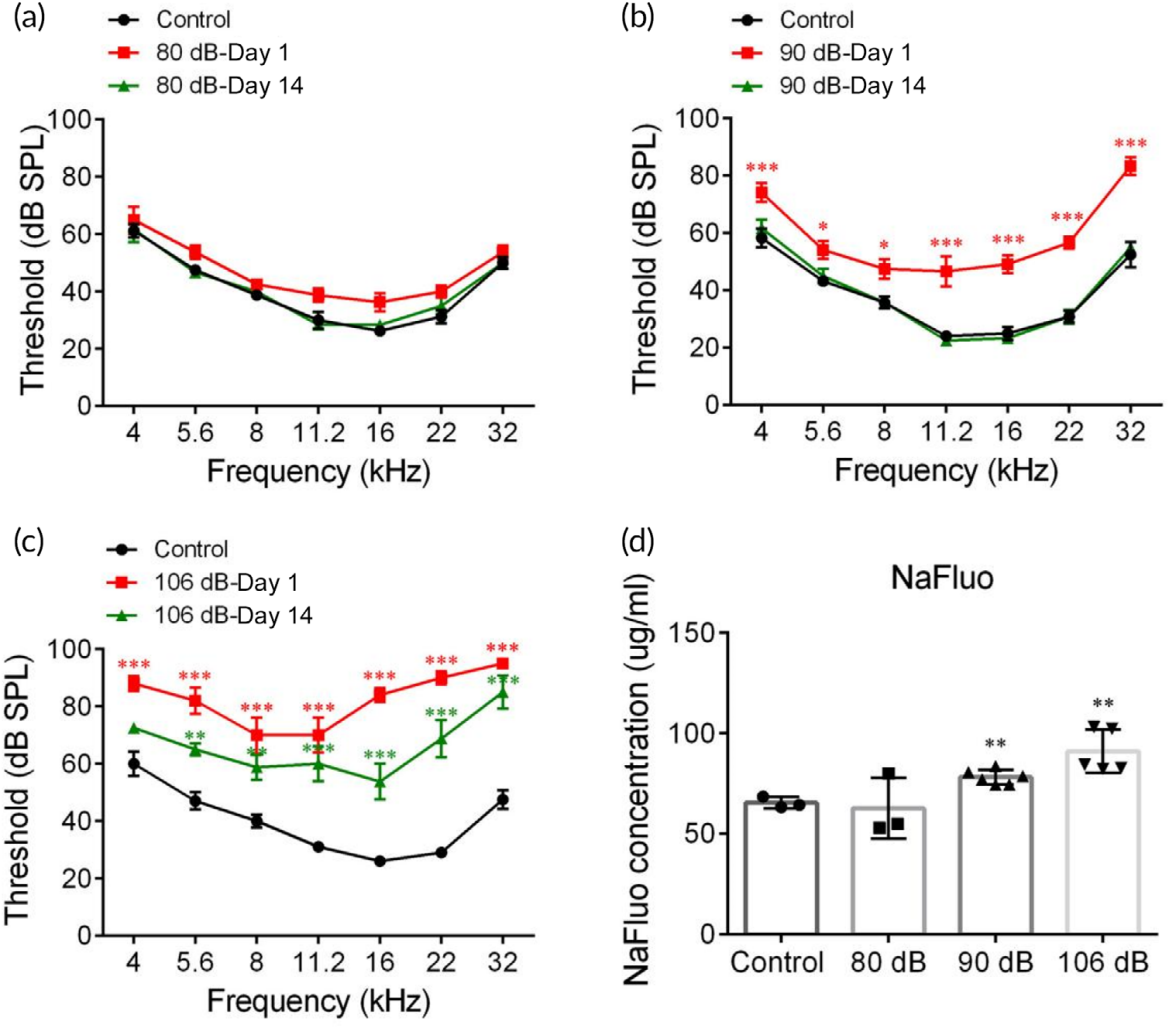
Screening for the optimal condition of sound exposure. Auditory brainstem response thresholds in control, Days 1 and 14 post various sound exposures, including 80 (a), 90 (b), or 106 (c) decibel sound pressure level (dB SPL) 8–16 kHz, 2 h (*n* = 5). (d) Concentrations of sodium fluorescein (NaFluo) calculated by a standard curve (*n* = 4). *p < 0.05, **p < 0.01 and ***p < 0.001 versus Control.

An 8–16 kHz octave‐band of sound has been frequently used as a sound exposure in noise‐induced hearing loss (NIHL) researches.^23^, ^24^, ^25^ Cochlear degeneration or injuries are dependent on sound intensities.^26^ Here, we screened the optimal condition of SC by exposing animals to 8–16 kHz octave‐band at different intensities for 2 h. 80, 90, and 106 dB SPL were selected as low, moderate, and high intensity of noise exposures. In the previous literature, 2 h of 106 dB SPL 8–16 kHz noise exposure has been commonly used to induce permanent hearing loss.^27^, ^28^, ^29^ We first examined the auditory thresholds 1 and 14 days after sound exposure of various intensities. 80 and 90 dB SPL‐conditioned mice demonstrated a reversible elevation in the ABR thresholds 14 days after sound exposure (Figure 1a,b), whereas 106 dB SPL might induce a permanent threshold shift (Figure 1c). We then evaluated the permeability of BLB by quantifying the fluorescence intensity of sodium fluorescein (NaFluo), which might to be the earliest and the most sensitive indicator of BLB disruption due to its low‐MW and being easy to detect at subtle concentrations.^30^ As illustrated in Figure 1d, the concentrations of NaFluo calculated by a standard curve (Figure S1) were significantly increased in 90 and 106 dB SPL groups, indicating an extravasation from blood to perivascular spaces in SV. However, 106 dB SPL was taken out of consideration on account of the permanent threshold shift (Figure 1c).

**FIGURE 2:**
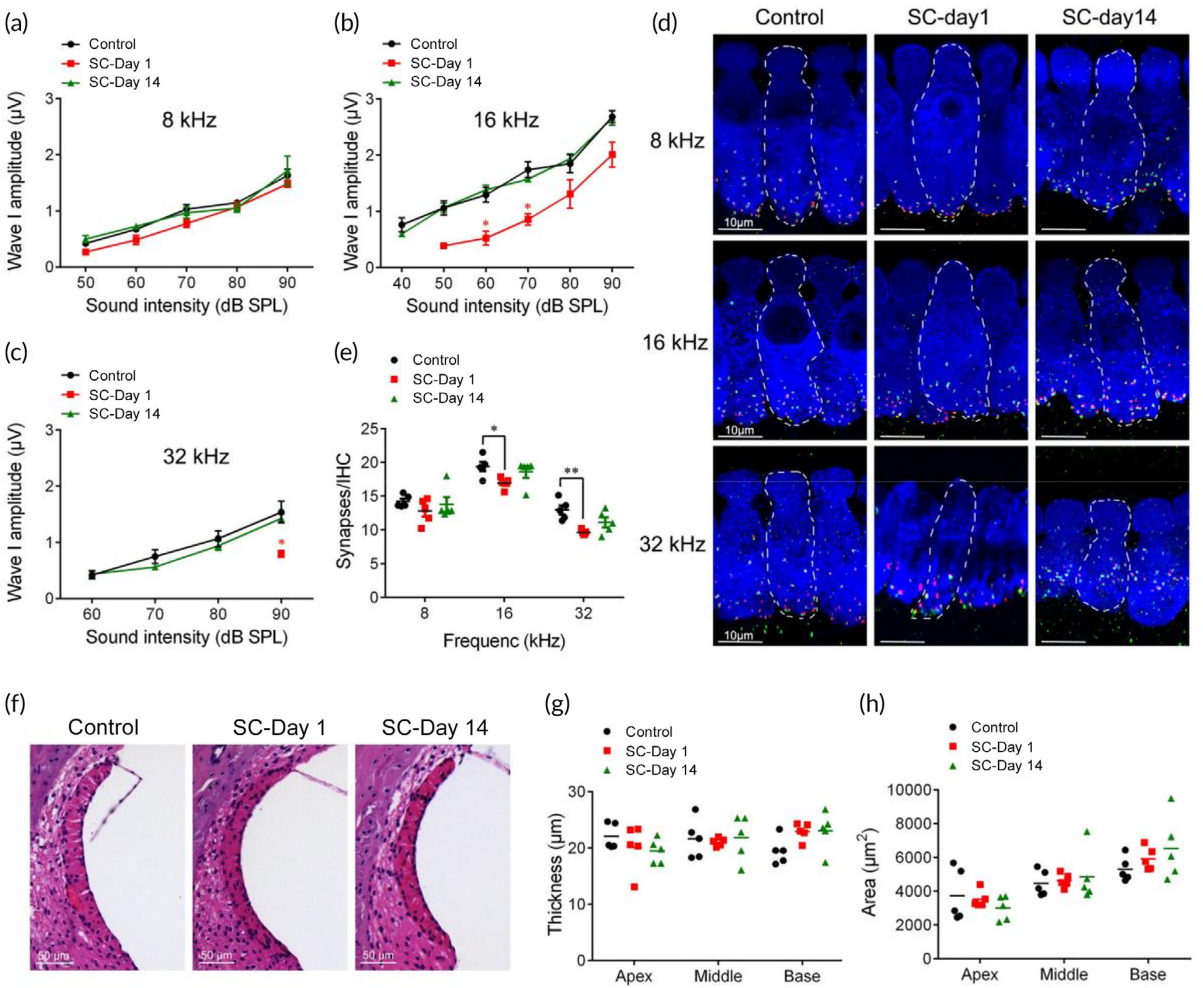
Safety evaluation of sound conditioning (SC). (a–c). Auditory brainstem response Wave‐I amplitudes of indicated frequencies (8, 16, and 32 kHz) in control, Days 1 and 14 post‐SC groups (*n* = 6). (d,e). Immunostaining and relative synaptic puncta counts for presynaptic ribbons (CtBP2, green), postsynaptic patches (GluA2, red), and inner hair cells (Myosin VIIa, blue) at 8, 16, and 32 kHz in control, Days 1 and 14 post‐SC groups (*n* = 5). (f). Representative images of SV in control, Days 1 and 14 post‐SC groups (*n* = 5). (g,h). Histological examinations of area and thickness of SV (*n* = 5). *p < 0.05, **p < 0.01 versus Control.

It is further explored whether SC resulted in pathological damages in organ of Corti's or SV by measuring ABR amplitude of the first peak (Wave‐I), immunostaining of synapses or outer hair cells (OHCs) and H&E staining of SV. The potential of synapse injuries should be taken into consideration in the applications of any conditions of sound exposures. Wave‐I amplitude represents the summed activity of the cochlear afferent nerves and the number of synapses is proportional to that of synchronously spiking SGNs.^31^ In Figure 2a–c, ABR Wave‐I amplitudes reduced at Day 1 post‐SC and recovered to near control values after 14 days at middle to high frequencies. Correspondingly, the number of synaptic puncta, immunolabeled by CtBP2 and GluA2 for pre‐synaptic ribbon and postsynaptic receptor patches, was reduced 1 day after SC and partially recovered by 14 days (Figure 2d,e). Besides, to evaluate the alterations in organ of corti and SV induced by SC, we immunostained OHCs in indicated frequencies (8, 16, and 32 kHz; Figure S2A) and observed the morphology of SV (Figure 2f). Results showed that no significant difference in the OHC counting and cross‐sectional area or thickness was found between control and sound conditioned mice (Figure 2g,h, Figure S2B).

Therefore, these results suggest 90 dB SPL as an appropriate sound intensity for BLB hyperpermeability and biologically safe for use.

### 3.2 Evaluation of paracellular and transcellular permeability

**FIGURE 3:**
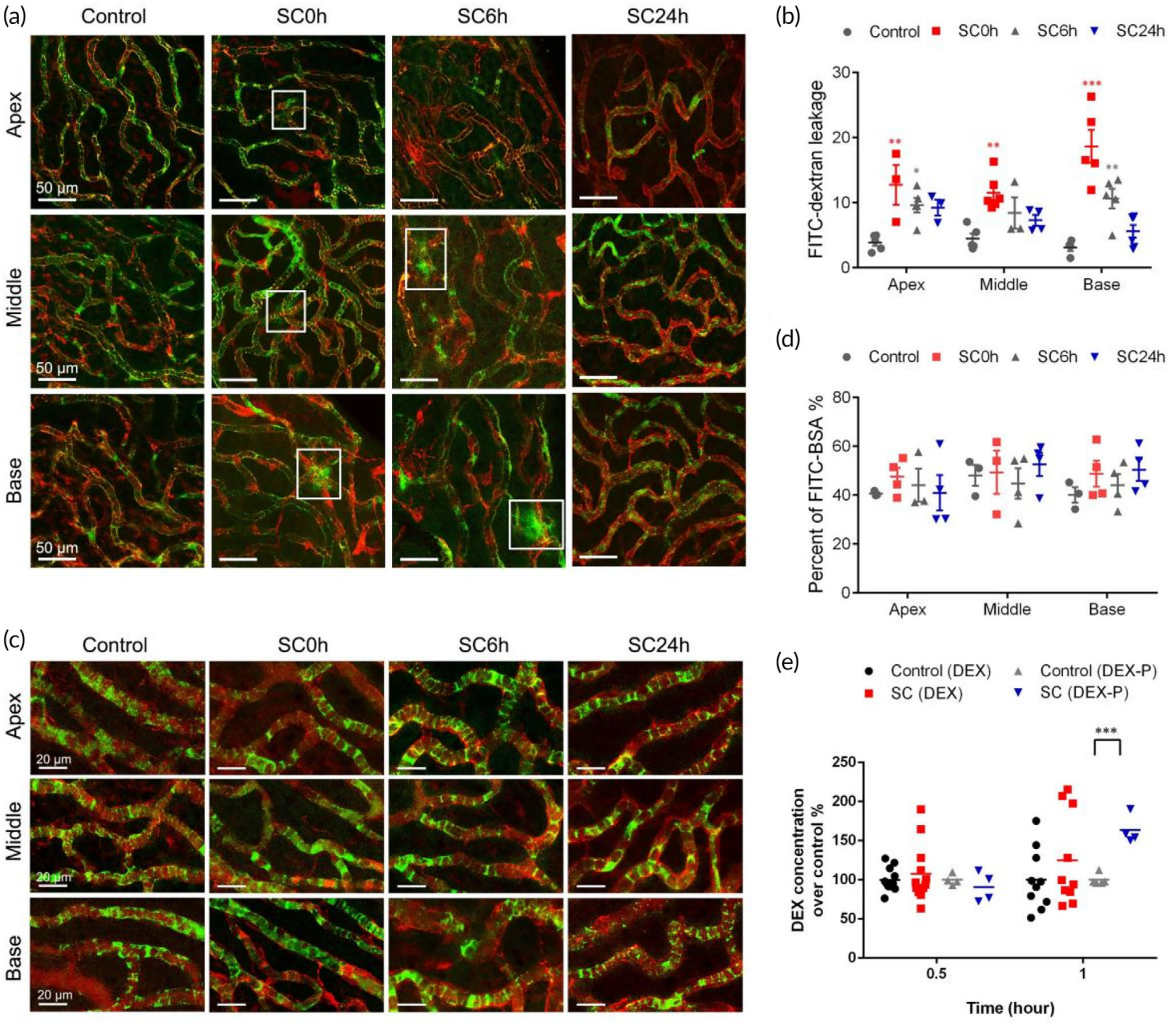
Evaluation of blood‐labyrinth‐barrier permeability in paracellular and transcellular pathway. The blood‐labyrinth barrier permeability for FITC‐dextran (a) and FITC‐BSA (c) in groups of control and 0, 6, and 24 h after sound conditioning (SC) in apical, middle, and basal turns. (b) Quantifications of FITC‐dextran leakage. (d) The percentage of the endothelial area filled with FITC‐BSA. (e) Cochlear concentrations of DEX and DEX‐P in control and SC groups (*n* = 5). *p < 0.05, **p < 0.01, and ***p < 0.001 versus control.

Transcellular and paracellular transport are two major pathways involved in regulating an appropriate delivery of fluids and solutes across microvascular endothelial barriers. The potential pathway underlying the enhanced permeability of BLB induced by SC was assessed by fluorescent tracers (FITC‐dextran and FITC‐BSA) at various time points (0, 6, and 24 h) after SC. FITC‐dextran (MW: 70 kDa) was used for evaluation of paracellular permeability of BLB, with GS‐IB4 visualizing the microvessels. As shown in Figure 3a, green fluorescence puncta were seen externally to the vessels within 6 h post‐SC, indicating the strial capillary leakage. The intensities of extravascular FITC increased in the apical and basal portions of SC‐treated group, which lasted for 6 h post‐SC (Figure 3b), providing a potential time window for blood‐borne drug administration. We then analyzed the role of transcellular pathway after SC by trafficking FITC‐BSA (MW: 68 kDa) which crosses the barrier from the blood to the interstitium by endothelial cell caveolae. The fractions of GS‐IB4‐positive EC areas that were filled with FITC tracer conjugated albumin were unchanged after SC at all regions (Figure 3c,d). Taken together, the increased dextran extravasation in SV as early as 0 h post‐SC, suggests that paracellular transport but not transcytosis or endocytosis accounts for the initial impairment of barrier function and temporary hyperpermeability after SC. These results indicated that the time window for systemic drug delivery was 0–6 h post‐SC and the molecular size limit of solutes that can penetrate through BLB was determined to be below 70 kDa, with respective hydrodynamic diameters of about 10.2 nm (^32^).

To verify whether permeability of BBB was enhanced by SC, EB dye was intravenously injected. No extravasation of EB staining was observed in brain tissues after SC (Figure 3), indicating no damage in BBB by SC.

### 3.3 Applications for drug delivery

Glucocorticoids (GC) are clinically used to treat Meniere's disease and idiopathic sudden sensorineural hearing loss due to its ability to attenuate immune and inflammatory responses, improve cochlear blood flow, and coordination of ion balance, and so forth.^33^, ^34^ The small lipophilic molecule DEX, as a synthetic GC, is readily transported via lipid‐mediated free diffusion through biological barriers, whereas the hydrophilic form DEX‐P is transported by paracellular pathway.^35^, ^36^ Based on the result that paracellular pathway predominantly accounts for the hyperpermeability of BLB after SC, we hypothesize that SC benefits the systemic delivery of DEX‐P into the cochlea rather than DEX. Here we quantified the concentrations of DEX and DEX‐P in the inner ear (Figure 3e). After systemically circulating for 1 h, cochlear DEX concentration was slightly elevated (about 20% higher, *p* > 0.05) in SC group. Meanwhile, a significantly higher content of DEX‐P was detected in SC group (*p* < 0.001), about 70% higher than the control group, confirming the participation of paracellular pathway in SC‐induced hyperpermeability of BLB. However, the cochlear drug levels of either DEX or DEX‐P in 0.5 h post‐SC groups were not significantly enhanced compared with the control groups.

### 3.4 RNA‐sequencing for underlying pathways

**FIGURE 4:**
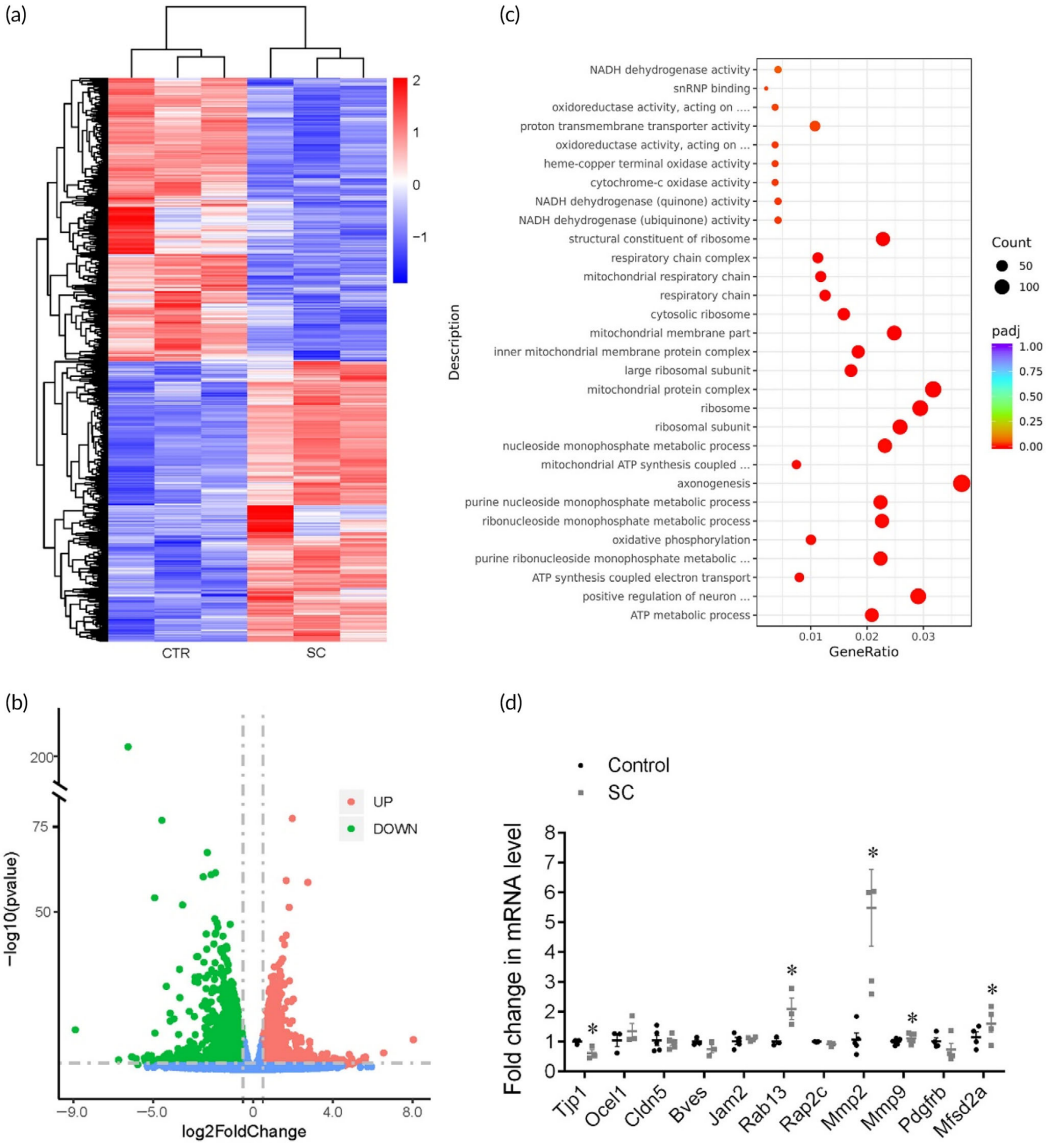
RNA‐sequencing for underlying pathways of sound conditioning (SC)‐induced BLB hyperpermeability. (a) Differentially expressed genes (DEGs) in the control and SC group. (b) Volcano plots depict differential gene expression of the control and SC group. (c) Gene ontology (GO) analysis. (d) qRT‐PCR of candidate genes (*n* = 4). *p < 0.05 versus control.

To explore the underlying mechanisms of SC‐induced BLB hyperpermeability, we detected the genes and pathways using a high‐throughput RNA‐sequencing of mice SVs from the control and SC groups (Figure 4a). A *p*‐value < 0.05 was used as the cutoff value for gene expression in the control and SC group to determine the DEGs. As a result, 2261 upregulated and 2237 downregulated DEGs in SV were identified after SC (Figure 4b). To investigate the potential pathways involved in the SC‐induced BLB hyperpermeation, DEGs were subjected to sequential GO analysis, in which several subterms related to respiratory chain or mitochondrial function (Figure 4c), indicating a stress response induced by SC. It is remarkable that TJ pathway (mmu04530) was found to be involved by KEGG analysis (Figure S4), with 16 upregulated genes and 18 downregulated genes.

Then, we identified 11 candidate genes: *Tjp1*, *Oclel1*, *Cldn5*, *Bves*, *Jam2*, *Cdh23*, *Rab13*, *Rap2c*, *Mmp2*, *Mmp9*, and *Mfsd2a* (Figure 4d). As expected, the qRT‐PCR assays corresponded with the RNA‐sequencing results. The level of TJ‐associated gene *Tjp1* was downregulated, whereas the level of *Oclel1* and *Cldn5* stayed unchanged. The levels of *Rab13*, the negative regulator of TJ assembly, and *Mmp2/9*, which degrade TJ proteins showed preferential elevation after SC. *Mfsd2a*, an inhibiting factor of vesicular trafficking to maintain BBB integrity, was downregulated in SC group.

### 3.5 Endothelial TJ changes underlying paracellular permeability

**FIGURE 5:**
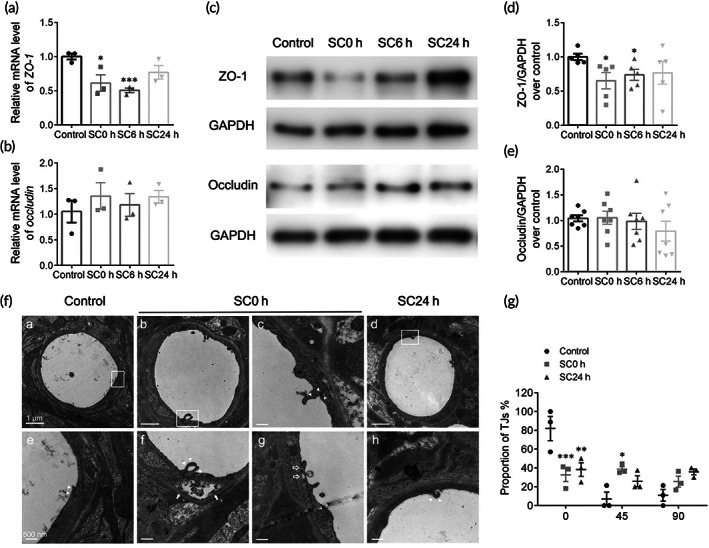
Mechanisms of paracellular pathway—tight junction changes. mRNA expression levels of *ZO*‐*1* (a) and *occludin* (b) in groups of control and 0, 6, and 24 h after SC (*n* = 5). Representative western blot images (c) and quantifications of ZO‐1 (d), and occludin (e). Protein bands were normalized to GAPDH (*n* = 5). (f) Representative transmission electron microscopy images of strial vessels and magnified versions (rectangle, below) of tight junctions (TJs) from control (a,e), SC0 h (b,c,f,g) and SC24 h (d,h) mice. (g) Fractions of angles of TJs. *p < 0.05, **p < 0.01, and ***p < 0.001 versus control.

TJs are indispensable for the integrity of BLB by avoiding paracellular transport. The major tight junction proteins in the BLB include zona occludens (ZOs), occludin, and claudins. Here, to assess whether the disruption of TJs appeared in BLB, we measured the mRNA and protein level of ZO‐1 and occludin, which have previously been identified in SV.^37^ ZO‐1 plays an integral role in TJ protein assembly and link TJ transmembrane proteins with the actin cytoskeleton, while occludin is associated with the formation of the intramembrane diffusion barrier.^38^ Compared with control mice, mRNA levels of ZO‐1 significantly decreased within 6 h post‐SC (Figure 5a). Consistently, western blotting results of ZO‐1 demonstrated obvious decline in SC and SC6h group (Figure 5c,d). Meanwhile, the mRNA and protein levels of occludin stayed still after SC (Figure 5b,c,e). Previous studies verified that occludin is not indispensable for the formation of TJ strands or function as a barrier.^39^, ^40^

Ultrastructural changes in BLB morphology after SC were examined by TEM (Figure 5f). Capillaries obtained from control group exhibited a smooth, continuous lining endothelium (Figure 5f(e), white arrowheads), while SC0 h mice showed an abnormal endothelium, including luminal endothelial protrusions (Figure 5f(f), white arrowheads), extensive separation of TJs, gapping in basement membrane (Figure 5f(f), white arrows) or TJs (Figure 5f(g), hollow white arrows), indicating the opening of TJs between endothelial cells. To quantify the rearrangement of TJs, we classify the angles of TJs as parallel to the lumen (0°), perpendicular to the lumen (90°) or in between (45°). As shown in Figure 5g, the majority of TJs were parallel to the vessel lumen in the control group, while SC altered the alignment of junctions, being parallel, perpendicular, or at various angles to the lumen. The fraction of parallel TJs was highly significant immediately after SC but not 24 h post‐SC, suggesting that the vascular leakage above was attributed to ultrastructural TJ abnormalities.

### 3.6 Rab13 inhibits the recruitment of ZO‐1

**FIGURE 6:**
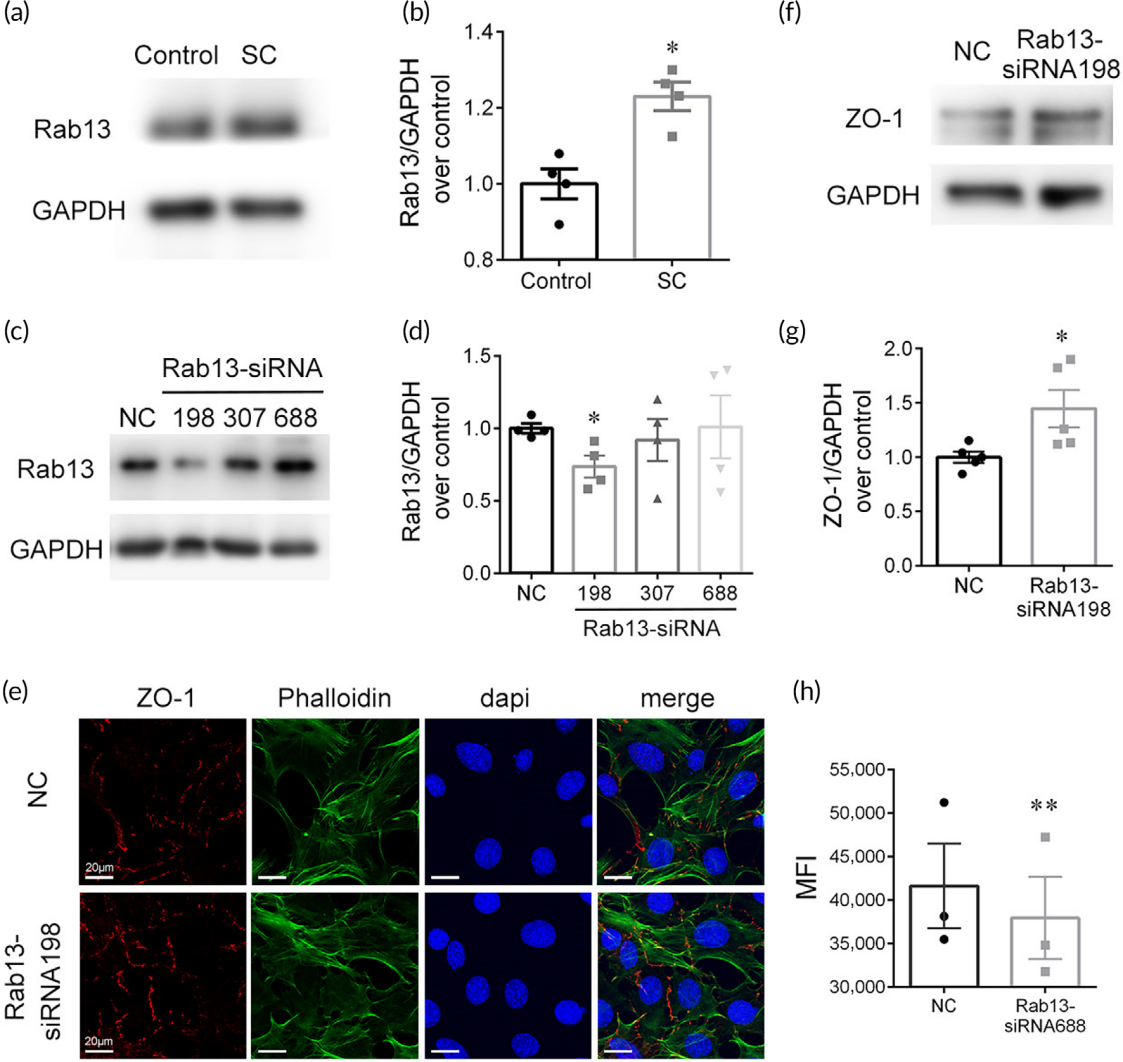
Rab13 inhibits the recruitment of ZO‐1. (a,b) Representative western blot images and quantifications of Rab13 in control and SC group (*n* = 5). (c,d) Screening an efficient *Rab13* siRNA duplex in HEI‐OC1 cells (*n* = 4). (e) Immunofluorescence signal of ZO‐1 in HEI‐OC1 cells after Rab13 silencing. (f,g) Representative western blot images and quantifications of ZO‐1 after Rab13 silencing (*n* = 5). (h) In vitro transwell assay after Rab13 silencing (*n* = 4). *p < 0.05, **p < .01 versus control.

The protein expression of Rab13 in SV was shown to increase after the SC‐induced BLB permeability enhancement and TJ breakdown (Figure 6a,b). To determine whether Rab13 was involved in the decrease of ZO‐1, thus enhancing BLB permeability, we examined the interaction between Rab13 and ZO‐1. *Rab13* was knocked down by transfecting HEI‐OC1 cells with three specific Rab13 siRNA duplexes for screening an efficient one. The qRT‐PCR and WB analyses showed that *Rab13* was silenced to 70% by siRNA‐mus‐198 compared with the negative control (Figure 6c,d). It is visualized that the immunofluorescence signal of ZO‐1 at the cell–cell interface was intensified after *Rab13* silencing (Figure 6e). The protein expression of ZO‐1 (Figure 6f,g) was shown to be upregulated in *Rab13* knockdown HEI‐OC1 cells, suggesting an inhibiting impact of Rab13 on TJ proteins. In addition, the transwell assay (Figure 6h) showed that *Rab13* silencing increased the in vitro monolayer cellular permeability, confirming the negative role of Rab13 in the regulation of cellular permeability.

### 3.7 PCs and PVM/Ms changes

**FIGURE 7:**
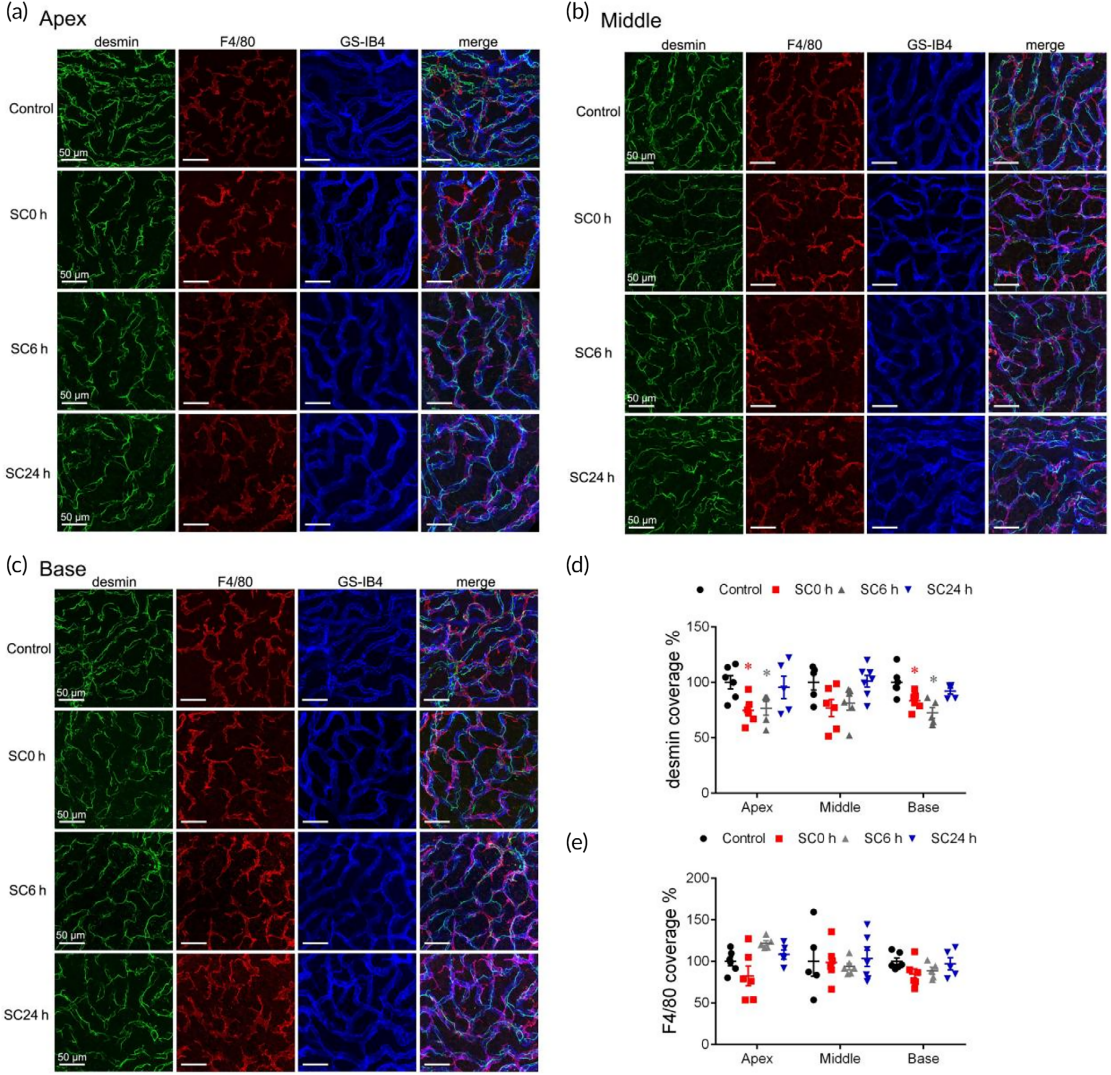
Mechanisms of paracellular pathway—PCs and perivascular resident macrophage‐like melanocytes (PVM/Ms) changes. (a–c) Confocal pictures of desmin‐positive PCs (green), F4/80‐positive PVM/Ms (red), and GS‐IB4‐positive microvessels (blue) in groups of control and 0, 6, and 24 h after sound conditioning (SC) in apical, middle, and basal turns of stria vascularis (SV). PC coverage (d) and PVM/M coverage (e) of the endothelium in control and 0, 6, and 24 h after SC (*n* = 4). *p < 0.05 versus control.

We next addressed the question of whether PCs and PVM/Ms changes accounts for BLB hyperpermeability. Previous studies showed that cochlear pericytes and PVM/Ms have an important role in maintaining barrier integrity.^4^, ^41^ Structural association between pericytes (with long processes that make contact with several endothelial cells along the long axis of the vessel), or PVM/Ms (with shorter process endings) and ECs are integral to the restrictiveness of the barrier.^42^ To determine whether SC regulates the expression of PC or PVM/M, we labeled them with desmin and F4/80 antibodies and calculated cell coverages (Figure 7a–c), quantified as the ratio of desmin or F4/80‐labeled area to isolectin GS‐IB4‐labeled area. Our results showed that pericyte coverage was reduced by about 25% in the capillaries of the SV within 6 h post‐SC (Figure 7d), however, there was no significant change on the PVM/M coverage (Figure 7e).

### 3.8 Transcellular pathway underlying BLB breakdown

**FIGURE 8:**
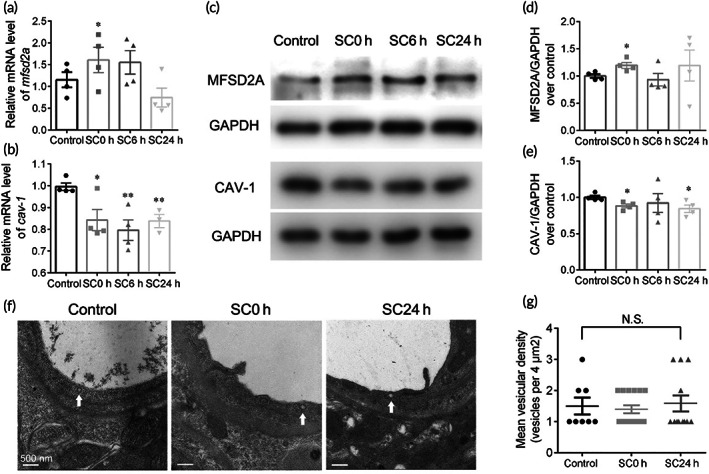
Mechanisms of transcellular pathway. mRNA expression levels of *mfsd2a* (a) and *cav*‐*1* (b) in groups of control and 0, 6, and 24 h after sound conditioning (SC; *n* = 5). Representative western blot images (c) and quantifications of MFSD2A (d) and CAV‐1 (e). Protein bands were normalized to GAPDH (*n* = 5). (f) Representative transmission electron microscopy images of cytoplasmic vesicles. (g) Quantifications of the number of vesicles per endothelial cross‐section. *p < 0.05, **p < 0.01 versus control. Not significant (N.S.).

Previous studies showed that major facilitator super family domain containing 2a (MFSD2A) was critical for BBB function by suppressing caveolae‐mediated transcytosis in CNS endothelial cells.^43^, ^44^ To examine the changes of MFSD2A after SC, we further analyzed mRNA and protein expressions by qRT‐PCR and western blot analyses in various time points, and found that mRNA and protein levels of MFSD2A were significantly increased instantly after SC (Figure 8a,c,d). Furthermore, the mRNA and protein levels of CAV‐1, as a major component and marker of the caveolae vesicles, were examined and shown to be reduced after SC (Figure 8b,c,e).

Since the irregular rearrangements of TJs correlating with the vascular leakage were observed after SC, we then examined the role of transcytosis in BLB hyperpermeability. In TEM analysis, the morphology of caveolae vesicle was shown as an electron‐dense circle with a hollow inside (Figure 8f, white arrows). Quantifications of vesicles showed a low level of vesicle trafficking in strial ECs and no significant changes occurred after SC (Figure 8g), in line with the unchanged fractions of FITC‐BSA trafficking (Figure 3c,d). The contradiction between the increased protein expression of CAV‐1 and steady number of caveolae vesicles may lie in the complexity in the regulation of caveolae formation, such as, other molecules belonging to caveolin family and the cavin complex.^45^ These results suggest a minor role of transcytosis in SC‐induced enhanced permeability.

## 4 DISCUSSION

Therapies for protecting, restoring, or regenerating hearing, including growth factors, stem cells, or viral vectors for gene therapy or optogenetics, are subject to biological barriers to delivery, primarily the BLB, which limits their clinical potential. Several methods have been investigated to overcome the anatomical barrier of BLB in the previous literatures, which could be exploited for enhancing the systemic delivery of therapeutics, including loop diuretics, inflammation, and acoustic trauma.^46^ For example, furosemide alters the permeability of the intrastrial fluid–blood barrier and enhances the distribution of the systemically injected drugs in the cochlea.^7^, ^8^ Moreover, the acute otitis media elevated the gentamicin uptake in the SV and sensory hair cells, probably attributing to the increased inflammatory cytokines that modulate the activity of endocytosis or selectively sensitize the expression of aminoglycoside‐permeant channels.^9^ Besides, noise exposure has been shown to cause ultrastructural changes in BLB and a specific decrease of TJs between adjacent endothelial cells, including ZO‐1, claudin‐5, and occludin,^13^, ^14^ which could be attributed to the reduced cochlear blood flow or inflammatory response.^47^, ^48^ Interestingly, a reversible opening of the BBB was found in mice by exposure to music below the safety threshold.^49^

The preconditioning effect is an interesting phenomenon, which refers to a toughening process making the cochlea more resistant to the subsequent detrimental forms of trauma. Several types of preconditioning have been studied, including hyperthermia, restraint, hypoxia, and sound.^16^ SC has been proven to stress the inner ear without causing permanent damage and resist against hearing loss.^50^, ^51^, ^52^ The possible mechanisms might involve F‐actin modulation^53^; apoptosis suppression^54^; stress responses, including heat shock proteins and GC^52^; increase in antioxidant enzyme activity and protection against reactive oxygen species.^55^ A study on young human showed that subjects exposed to a low‐level acoustic stimulation developed some resistance to noise‐induced temporary threshold shift.^21^ Recently, a clinical trial (NCT03878875) explored whether SC could reduce the temporary hearing loss and temporary tinnitus, although the results are not publicly available. To date, SC provides a potential strategy against ototoxic drug or acoustic trauma‐induced hearing loss; however, the underlying physiological mechanisms and appropriate interventions need further explorations.

This study first revealed that SC, in addition to toughening effect, could enhance the paracellular permeability of BLB and promoted the drug delivery into the inner ear (Figure 3). Notably, the superiority of SC over other regulatory factors is its high bioavailability, which holds a great promise for clinical translation. Apart from the simplicity in administration, SC‐induced hyperpermeability of the BLB was shown to be safe for auditory function without causing permanent hearing loss or cochlear synaptopathy after 2 weeks' restoration (Figure 2), as well as brain function without causing extravasation of BBB (Figure S3). Further studies are necessary to explore the protective effect of SC associated with blood‐derived drug therapy, acting as a toughening process on cochlea as well as providing an enhanced drug delivery, against sensorineural hearing loss. However, the preconditioning of sound also has limitations. SC enhanced the permeability of BLB in an attempt to augment substance delivery into the inner ear, but also let in circulating toxic substances that are normally excluded by the BLB. The increased risk of the toxic substance influx in the hyperpermeation time window induced by SC should be taken into account in the future clinical applications.

It is acknowledged that the pathway by which an exogenous drug cross physiological barriers depends on its basic physical characteristics, such as the simple “rule of five,”^56^ which are (1) MW < 500 Da, (2) high lipid solubility (<5 hydrogen bond donors, <10 hydrogen bond acceptors), and (3) the calculated Log P (CLogP) < 5. Drugs satisfying these requirements are likely to transport through BLB by transcellular lipophilic diffusion pathway. If not, passive paracellular or carrier‐mediated/receptor‐mediated transport is considered according to physicochemical parameters of drugs.^57^ Besides, interactions between drugs and transmembrane transporters are key determinants. Tahera et al.^52^ reported that an SC stimulus (8–16 kHz, 89 dB, 15 min) elevated plasma corticosterone with a consequent upregulation of glucocorticoid receptors (GR) in the cochlea. It is not known whether the increased GR contributes to an enhanced endocytosis of dexamethasone, so that the 70% elevation of drug distribution in cochlea (Figure 3e) could be the result of both paracellular diffusion and receptor‐mediated transport.

The investigations of mechanisms underlying the BLB hyperpermeability after SC clarified the paracellular transport as the predominant role, rather than transcellular pathway (Figures 4, 5, and 8). TJ members, *Tjps*, *Cldns*, *and Jams*, and so forth, act as gates of the paracellular diffusion as well as fences keeping the identity of plasma membrane domains.^58^ RNA‐seq, qRT‐PCR, and WB analyses determined the major role of the downregulation of ZO‐1, a scaffolding protein, which connect transmembrane proteins to the actin cytoskeleton, in the hyperpermeability of BLB induced by SC. Matrix metalloproteinases, which have been reported to degrade ZO‐1 and further lead to breakdown of the BLB in NIHL (^14^), were significantly increased after SC. ZO‐1 connects transmembrane proteins to the underlying actin cytoskeleton and recruit cytosolic proteins, such as kinases (e.g., GTPases) to TJs. Rab proteins, a family of small GTPases, play an important role in TJ assembly by recruiting TJ proteins from a cytoplasmic pool to cell–cell contacts.^59^ Rab13 has been verified to inhibit TJ integrity by decreasing the direct interaction between ZO‐1 and occludin accompanied with an increment in protein kinase A activity.^60^, ^61^ The level of Rab13 was significantly elevated after SC and knockdown of *Rab13* was verified to enhance the barrier permeability (Figure 6), indicating that the negative regulatory impact of Rab13 on TJ recruitment contributes to the breakdown of the BLB integrity.

Moreover, our results demonstrated that moderate sound exposure reduced the coverage of the pericyte structural protein desmin over the vessels in SV (Figure 7). Pericyte‐deficient mice have been shown to lead to BBB leakage due to TJ disruption.^62^ Similarly in cochlea, it has been reported that pericytes regulate TJ formation by signaling to endothelial cells.^63^ Collectively, the downregulation of ZO‐1 might be an integrated result of the increased degeneration of MMPs, the enhanced negative impact of Rab13 on TJ assembly, and the reduced coverage of pericyte around capillaries. However, more studies are needed to confirm the direct interactions between TJs and the factors above.

## 5 CONCLUSION

SC (90 dB SPL, 8–16 kHz, 2 h) enhanced the permeability of the BLB and provided a time window (0–6 h) for substances in the vasculature to readily enter the inner ear by paracellular pathway, characterized by disruptions in TJs. SC is a safe, clinically applicable, minimally invasive method for systemic pharmaceutical delivery to treat inner ear disorders.

## AUTHOR CONTRIBUTIONS

**Xueling Wang:** Project administration (equal). **Jiayi Gu:** Formal analysis (equal); writing – original draft (equal). **Baoying Xu:** Methodology (equal); validation (equal). **Ke Xu:** Methodology (equal); validation (equal). **Dehong Yu:** Conceptualization (equal); writing – review and editing (equal). **Hao Wu:** Funding acquisition (equal); resources (equal).

## FUNDING INFORMATION

This study was supported by the National Natural Science Foundation of China (Nos. 81970874 and 81970872), Natural Science Foundation of Shanghai (No. 21ZR1437600), Shanghai Municipal Science and Technology Major Project (Nos. 21JC1404000 and 2018SHZDZX05), Cross‐disciplinary Research Fund of Shanghai Ninth People's Hospital, Shanghai JiaoTong university School of Medicine (JYJC202231), and Shanghai Key Laboratory of Translational Medicine on Ear and Nose Diseases (No. 14DZ2260300).

## CONFLICT OF INTEREST STATEMENT

The authors declare no conflict of interest.

### PEER REVIEW

The peer review history for this article is available at https://www.webofscience.com/api/gateway/wos/peer-review/10.1002/btm2.10596.

## Supporting information


**FIGURE S1.** A standard curve of NaFluo. This correlation is represented by the equation *y* = 469.82*x* + 880.57 (*n* = 8, *R* ^2^ = 0.997, *p* < 0.001).
**FIGURE S2.** Changes in organ of corti and SV. Representative confocal imaging (A) and quantifications (B) of hair cells (Myosin VII, green) from indicated regions (8, 16, and 32 kHz) of control or Days 1 and 14 post‐sound conditioning mouse cochlea (*n* = 4). (C) Representative images of SV in control, Days 1 and 14 post‐sound conditioning groups by H&E staining. (D,E) Histological examination of area and thickness of SV (*n* = 5)
**FIGURE S3.** Representative images of Evans blue dye in brain tissues.
**FIGURE S4.** KEGG analysis on TJ pathway (mmu04530).
**FIGURE S5.** Original data of western blot images.

## Data Availability

The data that support the findings of this study are available from the corresponding author upon reasonable request.
