# Supplementary material for: Sound conditioning strategy promoting paracellular permeability of the blood‐labyrinth‐barrier benefits inner ear drug delivery
Source: Bioeng Transl Med. 2023 Sep 10;9(1):e10596. doi: 10.1002/btm2.10596 (PMC10771554; doi:10.1002/btm2.10596)
Supplement: Supplementary file 1 — FIGURE S1. A standard curve of NaFluo. This correlation is represented by the equation y = 469.82x + 880.57 (n = 8, R 2 = 0.997, p < 0.001). FIGURE S2. Changes in organ of corti and SV. Representative confocal imaging (A) and quantifications (B) of hair cells (Myosin VII, green) from indicated regions (8, 16, and 32 kHz) of control or Days 1 and 14 post‐sound conditioning mouse cochlea (n = 4). (C) Representative images of SV in control, Days 1 and 14 post‐sound conditioning groups by H&E staining. (D,E) Histological examination of area and thickness of SV (n = 5) FIGURE S3. Representative images of Evans blue dye in brain tissues. FIGURE S4. KEGG analysis on TJ pathway (mmu04530). FIGURE S5. Original data of western blot images. [file BTM2-9-e10596-s001.docx]

SFig. 1. A standard curve of NaFluo. This correlation is represented by the equation y = 469.82x + 880.57 (n=8, R^2^=0.997, P<0.001).


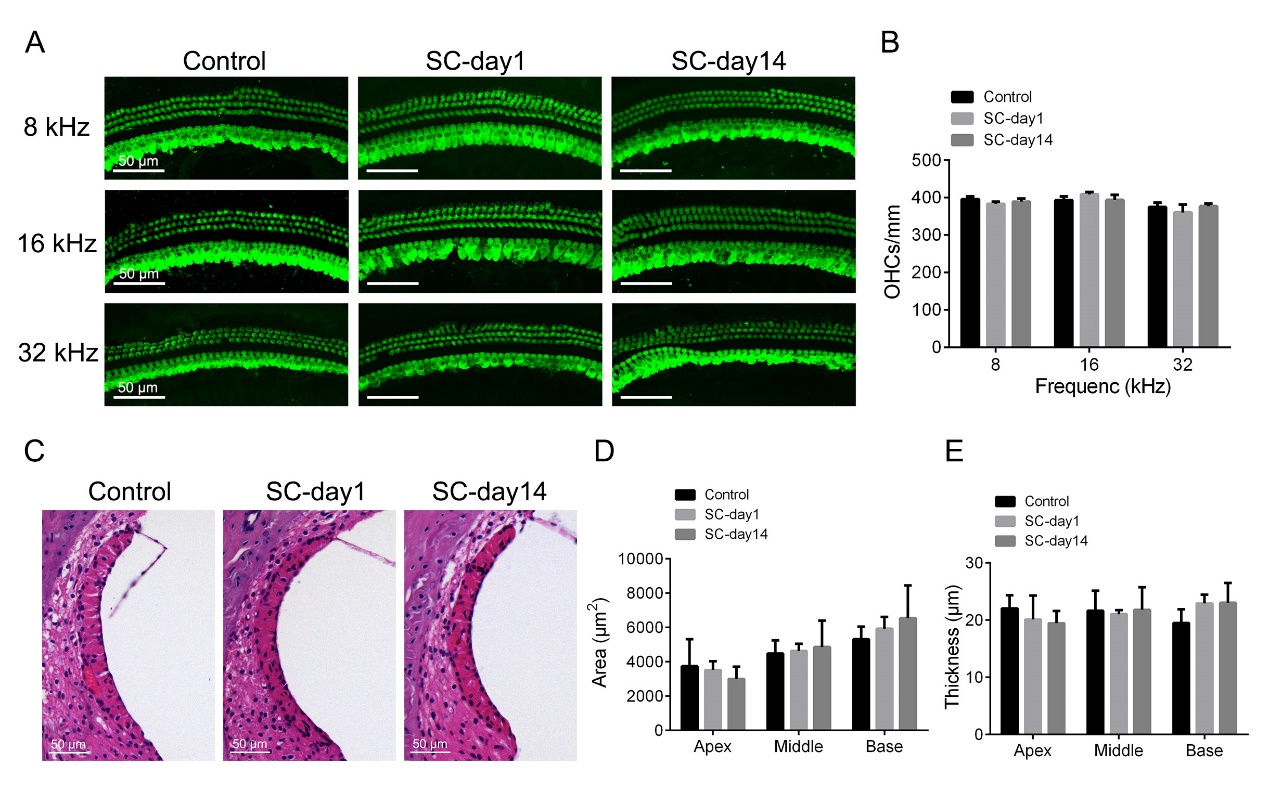


SFig. 2. Changes in organ of corti and SV.

Representative confocal imaging (A) and quantifications (B) of hair cells (Myosin VII, green) from indicated regions (8, 16, 32 kHz) of control or day 1, 14 post sound conditioning mouse cochlea (n = 4). C. Representative images of SV in control, day 1 and 14 post sound conditioning groups by H&E staining. D-E. Histological examination of area and thickness of SV (n = 5).


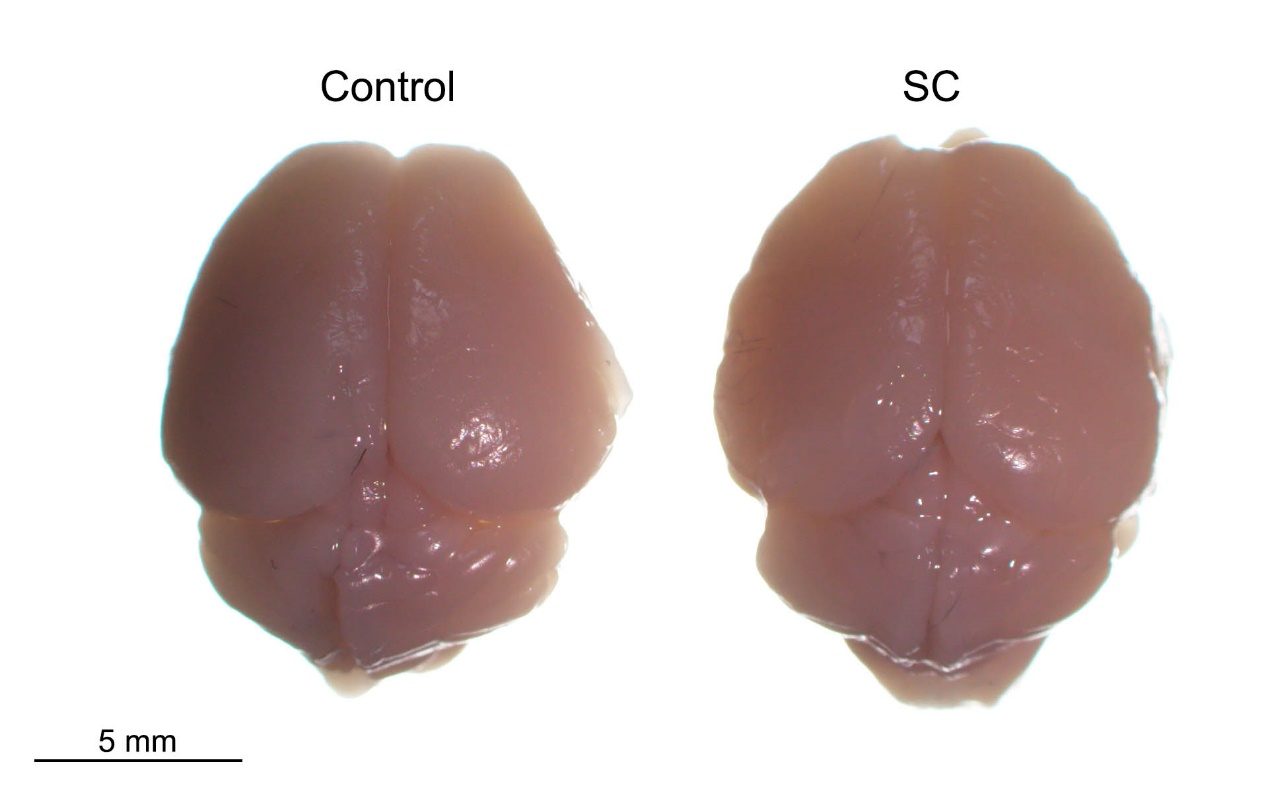


SFig. 3. Representative images of Evans blue dye in brain tissues.


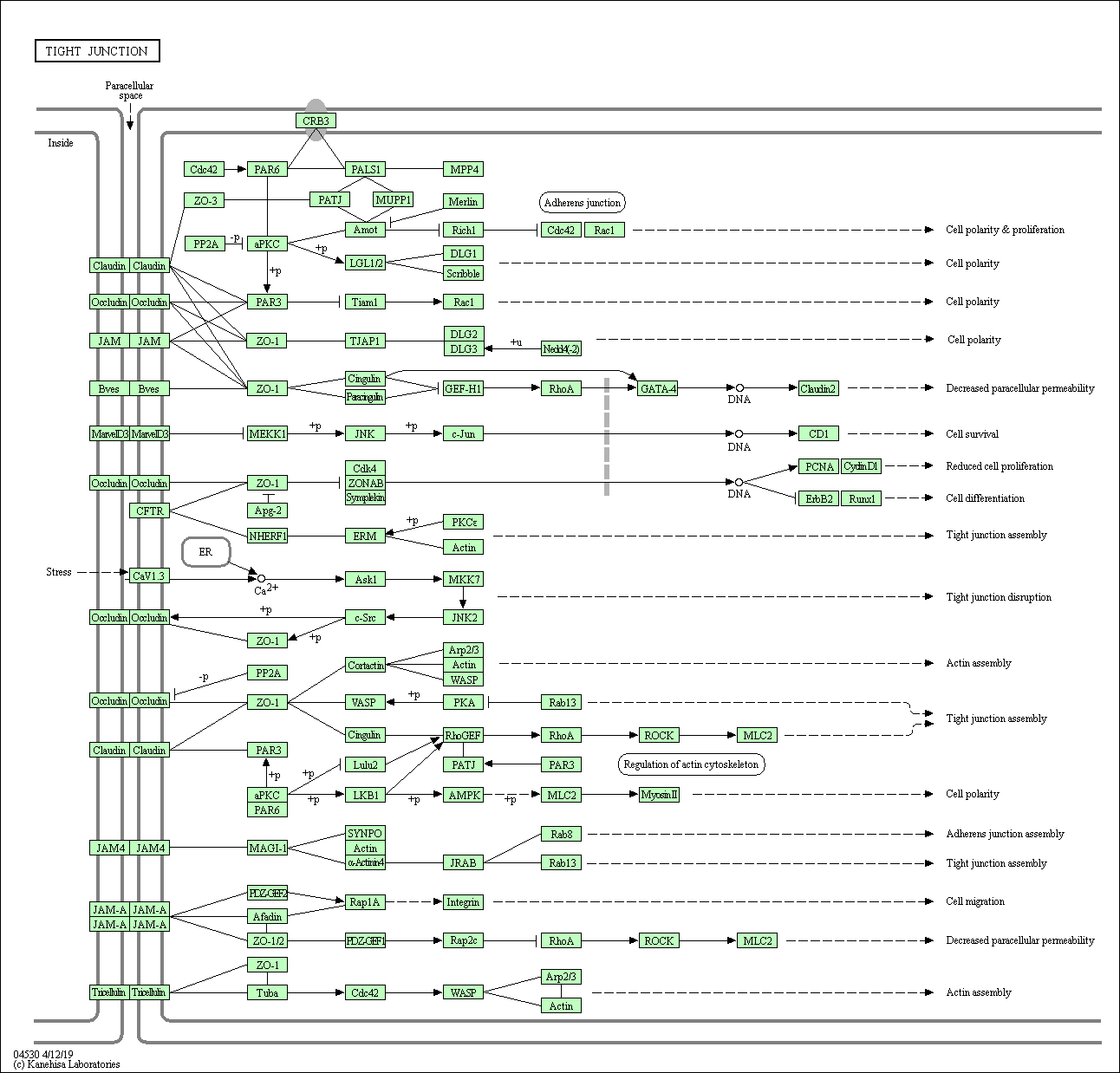


SFig. 4. KEGG analysis on TJ pathway (mmu04530).


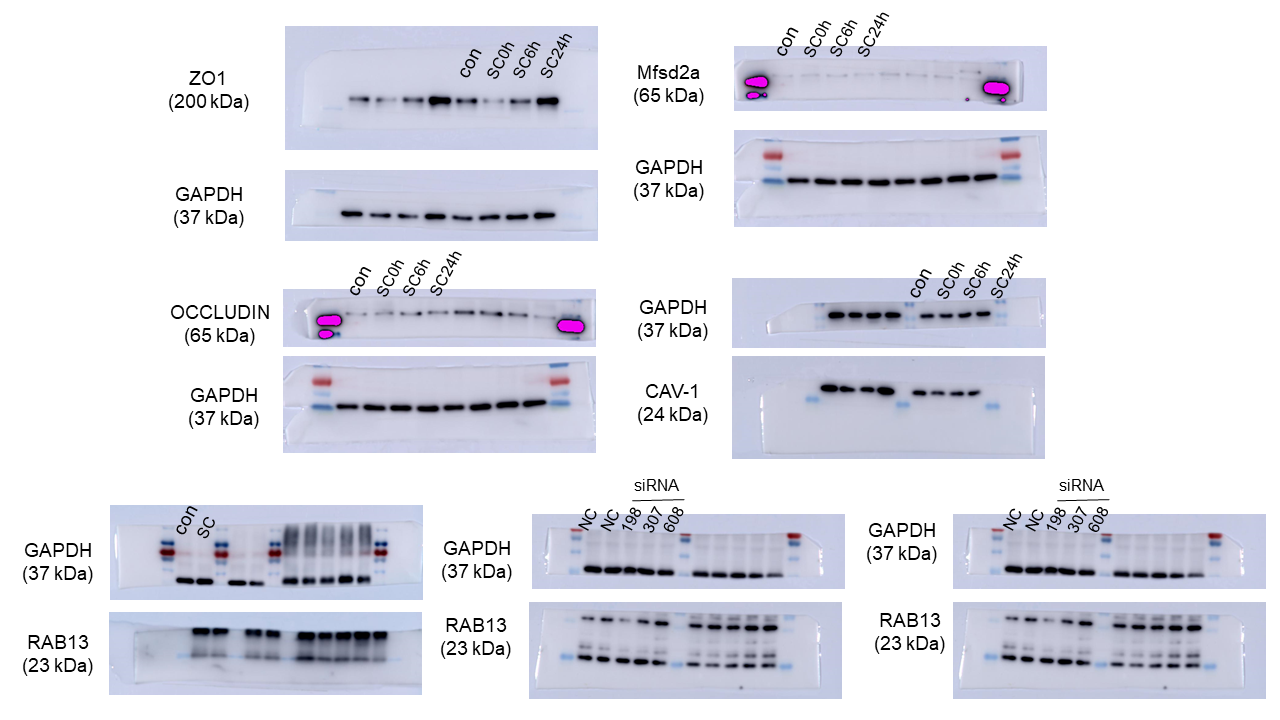


SFig. 5. Original data of western blot images.
